# Predicting food insecurity among UK university students during the COVID-19 pandemic

**DOI:** 10.1017/S1368980024001022

**Published:** 2024-05-03

**Authors:** Emily K Round, Sarah Weatherston, Paul B Stretesky, Margaret Anne Defeyter

**Affiliations:** 1 Healthy Living Lab, Faculty of Health & Life Sciences, Northumbria University, Newcastle-Upon-Tyne NE1 8ST, UK; 2 School of Social and Political Science, University of Lincoln, Brayford Pool, Lincoln LN6 7TS, UK

**Keywords:** Student food insecurity, Higher education, COVID-19, Food security

## Abstract

**Objective:**

The present study investigated potential predictors of food insecurity among UK university students during the COVID-19 pandemic.

**Design:**

Close-ended questionnaire administered to a cross-sectional sample of UK university students.

**Setting:**

Data were collected using an online survey platform in October 2020, during the COVID-19 pandemic.

**Participants:**

A nationally representative sample of UK university students (*n* 640).

**Results:**

Odds ratios (OR) obtained from logistic regression were statistically significant for three measures of economic hardship. First, students who relied on financial aid from student loans were 1·9 times more likely to report being food insecure than students who did not rely on financial aid from student loans. Second, students who could not pay their utility bill (*v*. those that could pay) were 3·1 times the odds of being food insecure. Finally, as perceived difficulty in paying for accommodation increased across the sample, the odds of being food insecure also increased (OR = 1·9). We also found that students who were recently ill were 2·2 times more likely to be food insecure compared with students who were not recently ill. We did not find any evidence that testing positive for COVID-19 predicted food insecurity, and university supplied food parcels/boxes did not reduce student food insecurity.

**Conclusions:**

Both economic factors and illness play a significant role in self-reported food insecurity in higher education students during pandemic lockdown. Further research is needed to explore food insecurity, economic factors and illness outside of a pandemic context.

Globally, the impact of COVID-19 altered many aspects of daily life, with approximately 664 million COVID-19 confirmed cases and 6·7 million deaths – numbers which are continually rising^(1)^. It is within the context of this global pandemic that students in UK higher education became increasingly isolated in their places of residence, with many unable to work, attend face-to-face university classes or socialise with people outside of their social ‘bubble’.

Given the reported impact of COVID-19 on student populations in the media, it is not surprising that the topic is starting to be researched^(2–4)^. A number of studies^(5–11)^ examine the correlates of food insecurity among higher education students during COVID-19 lockdown, yet there remains a paucity of research conducted in the UK. This research seeks to fill that gap in the UK literature by investigating those factors associated with food insecurity among a representative sample of UK university students during 2020.

## UK Universities and COVID-19

The COVID-19 pandemic reached the UK in January 2020. By March 2020, the British government began closing schools, the hospitality sector and non-urgent healthcare facilities. These restrictions were followed by the first national ‘lockdown’ which limited people’s movement. During this time, the UK government required the population to ‘stay at home’^(12)^. The restrictions were in full effect until July 2020 and resulted in the physical closure of many university campuses and the cancellation of almost all face-to-face teaching. However, many higher education students continued or started their course via remote learning^(13,14)^. Despite online efforts to the contrary, the UK government’s Office for Qualifications and Examinations Regulation^(15)^ reported that during the pandemic students were given less work, felt isolated and lacked access to appropriate resources such as study spaces, computers and the internet.

At the beginning of the national lockdown, 45 % of students continued living at their term time address^(16)^. Some students reported that they were prohibited from leaving their halls of residence by campus security^(17)^ and consequently experienced difficulties accessing food^(18)^. Whilst some universities provided food parcels, the nutritional quality of such parcels varied^(18)^; students reported that there was a lack of consideration for religious or dietary requirements and some universities charged for this service^(19)^. In response, some universities offered students a stipend to spend at local mobile food outlets that were invited to attend residences to sell their produce, whilst other universities sent communications detailing how students could access food and other provisions from local supermarkets^(17)^.

Regardless of endeavours by universities to help students access food, there was simultaneously a growing concern about the negative impact of restrictions on student health and well-being^(13)^. Moreover, students experienced trouble paying rent on their accommodation^(20)^. Around sixty universities in England agreed to refund rent or allow students to terminate their rental contracts, alongside reductions by some private landlords and agencies, although many other universities opted not to alter contracts or waiver any fees^(20)^. Student financial hardship was likely compounded by the nature of student employment, and the impact of the pandemic and national lockdown policies on the retail and hospitality sectors^(21)^.

## Food insecurity among university students

Food insecurity is ‘the inability to acquire or consume an adequate quality or sufficient quantity of food in socially acceptable ways, or the uncertainty that one will be able to do so’^(22)^. University students are one population at risk of high levels of food insecurity^(23–25)^. A multitude of demographic factors are found to be correlated with university student food insecurity. For instance, racial/ethnic minority students are more likely than non-racial/ethnic minority students to experience high levels of food insecurity^(26–28)^. University students from low-income households, or with a history of receiving free school meals, are more likely than those from more affluent households to be food insecure^(28)^. Moreover, students who are not employed^(29)^ and those in receipt of financial aid^(26,30)^ are more likely to have higher levels of food insecurity than those who are employed or not in receipt of financial aid.

Research on the correlation between gender and food insecurity is also notable. Findings suggest that female students are more likely to be food insecure than male students^(31,32)^, perhaps because (1) female students are found to be more likely than male students to be economically disadvantaged and therefore more likely to be food insecure as a result^(33,34)^ and (2) female students are found to be more likely than male students to be open about their finances and depend on parental financial support^(34)^.

Finally, student living arrangements can also influence levels of food insecurity among university students^(35)^. Students who live with their families during term-time or on-campus, as opposed to independently or off-campus, are most often found to report the highest levels of food security^(36)^.

## Student food insecurity during COVID-19

Research by the National Union of Students^(37)^ found that 60 % of surveyed students stated that their income declined during the COVID-19 pandemic, with 70 % of students saying they were unsure whether they could manage their finances. In addition, during 2020, one in three UK students said they had to cut back on food for financial reasons, and one in ten reported using food banks^(37)^. As a result, many students returned home mid-semester^(38)^. However, changes in housing and living arrangements do not necessarily result in improved levels of food security^(38)^. Changes in living arrangements were, however, among the strongest predictors of student food insecurity during the pandemic^(5,39)^.

The COVID-19 pandemic was also disruptive for student health, as students who tested positive for COVID-19 were reported to have heightened levels of anxiety, depression and food insecurity^(37,40)^. Indeed, food insecurity is associated with poorer mental well-being, concentration, academic performance and physical health^(23,41–43)^. As countries employed differing approaches in their response to the spread of COVID-19 within higher education, it is important for researchers to explore this topic within individual countries^(44,45)^.

## Methods

### Design

In March 2020, the UK government imposed a set of lockdown restrictions to stop the incidence of COVID-19. Most university students were sent home at the end of the 2019/20 academic year and subsequently asked to begin the 2020/21 academic year in physical isolation where possible: instead undertaking remote or blended forms of teaching and assessment^(46)^. At the start of the 2020/21 academic year (i.e. in late October 2020), online questionnaires for this study were distributed to higher education students across England, Scotland, Wales and Northern Ireland. As a result, the present study is cross-sectional and based on a questionnaire of students attending UK universities during the peak of the coronavirus (COVID-19) lockdown. These cross-sectional data obtained from the questionnaire were used to understand which variables, if any, predict student food insecurity.

Ethical approval was granted from the Ethics Committee at Northumbria University (ethics reference number: 22790). Students were sampled using the online survey platform *Prolific* (https://www.prolific.co). *Prolific* is an online survey platform that compensates each research participant with a small amount of money for answering questions developed by academics, businesses and non-profit organisations. The *Prolific* database currently consists of over 100 000 potential research participants who can be pre-screened according to a variety of personal characteristics, such as university student status^(47)^. In the present study, we asked *Prolific* to recruit a representative sample of students attending UK universities to better understand their experiences with food insecurity during the COVID-19 pandemic. *Prolific* selected a sample of UK higher education students (*n* 640) from a list of 4758 eligible UK higher education students in their database. All students who agreed to take part in the study received compensation (around £2 GBP on average) for their time to complete the questionnaire, which took an average of 7 min.

The present study investigated potential predictors of food insecurity among university students during the COVID-19 pandemic. To identify variables used to predict food insecurity, we relied upon existing literature. As noted previously, gender, race, ethnic status, year of study, economic disadvantage, student loans, parent aid, employment status, financial pressures and living arrangements have all been found to predict levels of food insecurity among students^(31–36)^. Thus, we included variables to measure these concepts in the present study of food insecurity during the COVID-19 pandemic. The present study also included two measures of coronavirus (something that has yet to be examined in the university student food insecurity literature) and one measure of university food provision in the form of food parcels/boxes that were given to students by some universities at the beginning of the 2020/21 academic year.

### Variables

#### Outcome variable

The outcome measure in this study was food insecurity which is defined as a dichotomous variable where students were classified as food secure (scored ‘0’) or food insecure (scored ‘1’). This variable was created from the six-item USDA Adult Food Security Survey Module (AFSSM) where students were asked about their experiences accessing food since the beginning of the 2020/21 academic year (approximately 30 days)^(48)^. Questions in the AFSSM asked about their access to food (e.g. cut the size of meals or skipped meals, ate less, worried about food or could not afford to eat balanced meals). The items on the AFSSM were used to produce a *Food Security Score* ranging from 0 to 6 according to the USDA coding criteria. In this study, students scoring between two and six points were classified by the AFSSM as experiencing ‘low or very low food security’ (i.e. we therefore categorised them as ‘food insecure’) and students who scored between 0–1 points were classified by the AFSSM as experiencing ‘high or marginal food security’ (i.e. we therefore categorised these students as ‘food secure’).

#### Predictor variables

The predictor variables in this study were organised into three broad areas and two indicators of illness. These three sets of variables believed to be correlated with student food insecurity were (1) student focused demographics, (2) education focused demographics and (3) economic factors. Demographic variables included *gender*, *age* and *ethnicity.* To measure *gender*, students were asked to report the gender they identified as (i.e. ‘female,’ ‘male’, ‘non-binary’, ‘third gender’ or ‘self-describe’). The variable was measured using a dummy variable approach by using the values of ‘1’ to indicate the presence of a category of gender (i.e. ‘female’, ‘male’, or ‘non-binary/third gender’) and ‘0’ to indicate the absence of that category. The categorical effects were estimated by comparing females and non-binary/third-gender participants to males (the omitted category). *Age* was measured in years. Finally, *ethnicity* was measured by employing the UK’s official ethnic categories. We again used a dummy variable approach and estimated the effect of being Asian, Black, mixed/multiple ethnicity and other ethnicity in comparison to white (the omitted category).

Educational related demographic variables included *international student status*, *enrolment status*, *year of study* and *living arrangements*. These variables were found to be related to student food insecurity in previous research^(36,49)^. *International student* was a dichotomous variable that indicated whether a student came from outside the UK (scored ‘1’) or was a UK resident (scored ‘0’). *Enrolment status* was also dichotomous and indicated part time students (scored ‘1’) or full time students (scored ‘0’). *Year of study* was an ordinal variable that measured students’ classification on a scale of 1–7. Categories in this variable included ‘foundation year’ (scored ‘1’), ‘first year’, ‘second year’, ‘third year,’ ‘fourth year undergraduate’, ‘post-graduate Masters’ and ‘post-graduate PhD’ (scored ‘7’). Finally, *living arrangements* measured whether the student lived alone, with other students, with family members/relatives and/or with other non-student relatives. *Living arrangements* was operationalised using a dummy variable approach where values of ‘1’ indicated the presence of a living arrangement and ‘0’ indicated the absence of that living arrangement. In multivariate regression models, categorical effects of living arrangements were estimated by comparing different arrangements to living alone (the omitted category).

Economic factors are often found to be correlated with food insecurity and include indicators of *free school meal history*, *employment status*, *financial aid from student loans*, *unable to pay utilities*, *difficulty paying accommodation* and *university food parcel/box*. *Free school meal* history was a dichotomous variable that was scored ‘1’ if a student reported they were eligible for means-tested free school meals in the year prior to enrolling in university as an undergraduate (otherwise scored ‘0’). *Employment status* was also dichotomous and scored ‘1’ if a student was employed part time or full time and ‘0’ if not employed. Students who said they relied heavily on financial aid from student loans to pay rent and purchase food were given the score of ‘1’ on *financial aid from student loans,* whilst those who did not rely on loans were assigned a score of ‘0.’ Students struggling financially were considered in two ways. First, students who were unable to pay for their utilities such as water, electricity and gas in the previous month were given the score of ‘1’ on *unable to pay utilities* (else ‘0’). Second, students who agreed more with the statement ‘I am finding it difficult to pay my rent or mortgage’ were considered more financially insecure. Responses for the variable *difficulty paying accommodation* were Likert in nature and ranged from ‘Strongly Disagree’ (scored ‘1’) to ‘Strongly Agree’ (scored ‘5’). Finally, we asked students if they received a food parcel or food box from their university during the beginning of the 2020/21 academic year (*food parcel/box*). Students who said they were given a parcel/box by the university were scored ‘1’ on *food parcel/box*, whilst those that did not say they received a parcel/box were scored ‘0’.

We measured the impact of coronavirus effects in two ways. First, we identified students who reported testing positive for COVID-19 during the past 30 days (*COVID-19 Positive*). This was a dichotomous variable so that those who reported testing positive were assigned a score of ‘1’, whilst those that did not report testing positive were assigned a score of ‘0.’ As an alternative to testing positive, we also examined self-reported illness. Many students reported that they were unable or did not have access to COVID-19 tests (or testing facilities) or may not have wanted to tell us they had COVID-19 because of the stigma associated with the virus^(50)^. Thus, we asked students if they were burdened with any significant illness during the past month. The variable *significant illness* therefore represented a dichotomous variable where students who reported having a significant illness were given a score of ‘1’ whilst those who did not report an illness were given a score of ‘0’. Importantly, these illnesses may or may not have been thought of as COVID-19 related. Moreover, not all students testing positive for COVID-19 will have had symptoms that they classified as significant illness. As a result, measuring both illness and COVID-19 was important.

### Analytic strategy

We firstly looked for potential bivariate associations between the predictor and outcome variables to see if any important patterns appeared. We summarised these bivariate relationships between food insecurity and predictors in two ways. First, for categorical variables (i.e. nominal/ordinal/dichotomous variables), we reported the percentage (and frequency) of students who were food secure/food insecure in each category of the predictor variable (i.e. in a contingency table type format). Chi-square was used to test the null hypothesis that the variables are independent. For the variable *Age*, we estimated the mean age for food-secure and food-insecure students. We compared these means using a *t*-test for independent samples. Next, we examined the simultaneous impact of all predictor variables on food insecurity using multivariate logistic regression (LR). In each set of regressions, we present Odds ratios (OR). In the case of dichotomous predictor variables, the OR can be interpreted as the odds that food insecurity will occur when the condition is present compared with the odds that food insecurity will occur when the condition is not present. For continuous variables, the OR in food security are estimated for a one-unit change in the predictors. The software *Stata V15* was used for all data analyses. Since the sample was largely representative of the general student population, we did not weight these data. Finally, we conducted alternative statistical analyses using ordinary least squares regression on the range of scores for the AFSSM as the outcome variable. The coefficients for the analyses are found in Appendix A. The purpose of this analysis was to determine if a different operationalisation of food security may have led to a different outcome, potentially questioning the validity of our findings.

## Results

### Sample characteristics

According to the UK Higher Education Statistics Agency (HESA), in the 2020/21 academic year, there were 281 higher education providers across the UK (i.e. England, Northern Ireland, Scotland and Wales). Students in the current study reported they were enrolled with 161 of those providers. Students participating in the survey were more likely to be undergraduates (93 %) than those in the population, where 73 % of the students in the UK were undergraduates in 2020/21. The remainder of the student sample was relatively reflective of the total UK population of students. In particular, 65 % were female (*v*. 57 % in the population in 2020/21), 75 % were white (*v*. 74 % in the population in 2020/21), 42 % were under 21 years of age (*v*. 38 % in the population in 2020/21) and 20 % said they had received means-tested free school meals during secondary education (*v*. 19 % in the UK population in 2020/21). Importantly, Office for National Statistics Student Covid Insights Survey (Pilot 3) suggested that as of November 2020, ‘6 in 10 students’ reported that their learning was mainly desk-based (i.e. self-paced or online learning through lectures or tutorials) whilst they were enrolled in university. This estimate is comparable to our estimate in that 55 % of all students said they did not receive any face-to-face teaching on their university campus. In short, sample participants tended to mirror the general population in terms of basic demographics such as race and socio-economic status but were slightly more likely to be undergraduate and female than the general student population.

### Bivariate results

First, we examined bivariate results for predictor variables and student food insecurity. These results are presented in Table 1 and compare frequency distributions for students who say they are food secure (*n* 416 or 65 %) to frequency distributions for students who say they are food insecure (*n* 209 or 32·7 %). As Table 1 suggests, some interesting patterns appear in these data.

**Table 1:** Characteristics of 640 UK university students according to food security status, October 2020

Variable	Food Secure		Food Insecure		*P*[χ2]^†^	*P*[*t*]^‡^	
	%	*n*	%	*n*			
Participants, % (*f*)	65·0 %	416	32·7 %	209		–	–
Missing values	2·3 % (*n* 15)						
Gender, % (*f*)						0·43	
Female	65·3 %	264	34·7 %	140	100 %		
Male	69·7 %	145	30·3 %	63			
Third Gender/Non-Binary/Other	55·6 %	5	44·4 %	4			
Missing values	50·0 %	2	50·0 %	2			
Ethnicity, % (*f*)						0·28	
White	65·2 %	306	35·8 %	163	100 %		
Asian	71·4 %	60	28·6 %	24			
Black	81·3 %	26	18·8 %	6			
Mixed	62·1 %	18	37·9 %	11			
Other	55·6 %	5	44·4 %	4			
Missing values	50·0 %	1	50·0 %	1			
Year of study (scored 1–7), % (*f*)						<0·05^*^	
Foundation year	42·9 %	3	57·1 %	4	100 %		
1st Year	62·9 %	73	37·1 %	43			
2nd Year	72·2 %	135	27·8 %	52			
3rd Year	60·9 %	117	39·1 %	75			
4th Year	82·4 %	56	17·6 %	12			
MA post-graduate	52·5 %	21	47·5 %	19			
PhD post-graduate	80·0 %	4	20·0 %	1			
Missing values	66·5 %	7	33·5 %	3			
Living arrangements, % (*f*)						0·06	
Alone	59·7 %	37	40·3 %	25	100 %		
Other students	62·2 %	203	35·8 %	113			
Family	72·4 %	173	27·6 %	66			
Other	42·9 %	3	57·1 %	4			
Missing values	0	0	100 %	1			
Difficulty paying accommodation, (scored 1–5) % (*f*)						<0·05^*^	
Strongly disagree	83·2 %	153	16·8 %	31	100 %		
Disagree	78·8 %	145	21·2 %	39			
Neither agree nor disagree	63·4 %	59	36·6 %	34			
Agree	35·0 %	43	65·0 %	80			
Strongly agree	30·0 %	9	70·0 %	21			
Missing values	63·6 %	7	36·4 %	4			
International student, % (*f*)						0·21	
Yes	74·1 %	43	25·9 %	15	100 %		
No	66·0 %	370	34·0 %	191			
Missing values	50·0 %	3	50·0 %	3			
Part time student, % (*f*)						0·69	
Yes	64·6 %	53	35·4 %	29	100 %		
No	67·1 %	359	32·9 %	176			
Missing values	50·0 %	4	50·0 %	4			
Past free school meals, % (*f*)						<0·05^*^	
Yes	58·9 %	76	41·1 %	53	100 %		
No	68·7 %	340	31·3 %	155			
Missing values	0 %	0	100 %	1			
Employed, % (*f*)						0·61	
Yes	67·6 %	121	32·4 %	58	100 %		
No	65·4 %	282	34·6 %	149			
Missing values	86·7 %	13	13·3 %	2			
Financial aid from student loans, % (*f*)						<0·05^*^	
Yes	61·4 %	243	38·6 %	153	100 %		
No	75·0 %	168	25·0 %	56			
Missing values	100 %	5	0·0 %	0			
Unable to pay utilities, % (*f*)						<0·05^*^	
Yes	33·3 %	17	66·7 %	34	100 %		
No	69·6 %	392	30·4 %	171			
Missing values	63·6 %	7	36·4 %	4			
University food parcel/box, % (*f*)						0·58	
Yes	71·0 %	22	29·0 %	9	100 %		
No	93·5 %	389	95·2 %	199			
Missing values	1·2 %	5	0·5 %	1			
Covid-19 positive, % (*f*)						0·61	
Yes	3·8 %	16	4·3 %	9	100 %		
No	66·2 %	394	33·8 %	199			
Missing values	85·7 %	6	14·3 %	1			
Significant illness, % (*f*)						<0·05^*^	
Yes	56·1 %	101	43·9 %	79	100 %		
No	70·6 %	312	29·4 %	130			
Missing values	100 %	3	0 %	0			
Age (in years)							0·87
Mean	23·1		23·4				
sd	416		208				
Median	21		21				
Range	18–68		18–58				
Missing values	0 %	0	0·5 %	1			

* Reject null hypothesis, *α* level = 0·05.

† *P* value for χ2 test of independence.

‡ *P* value for two sample *t* test, 2-sided.

However, in these data food security/insecurity depended on *year of study* X^2^ = 19·4, 5 df, *P* < 0·05). On the one hand, 57 % (or four in seven) of foundation year students were food insecure, 47·5 % (or 19 in 40) of MA post-graduates were food insecure, 39·1 % (or 75 in 192) of 3rd year students were food insecure and 37·1 % (or 43 in 116) of first year students were food insecure. On the other hand, 27·8 % (or 52 in 187) of second year students were food insecure, 17·6 % (or 12 in 68) of fourth year students were food insecure and 20 % (or one in five) of PhD students were food insecure.

Students who had a history of receiving financial aid, relied heavily on student loans, were unable to pay utilities and/or agreed that they were struggling to pay for their accommodation reported higher mean food insecurity scores than those students who did not rely on financial aid from student loans or faced past or present financial hardship. First, 41·1 % (or 53 in 129) of all students who received means-tested free school meals in the past were food insecure compared with 31·3 % (or 155 in 495) who had not received free school meals (X^2^ = 4·6, 1 df, *P* < 0·05). In addition, 38·6 % (or 153 in 396) of students who relied on financial aid from student loans said they were food insecure, whilst 25 % (or 56 of 224) who did not receive financial aid from student loans were food insecure (X^2^ = 11·9, 1 df, *P* < 0·05). In addition, a large proportion of students (i.e. 66·7 % or 34 in 51) who were unable to pay their most recent utility bill were food insecure whilst a much smaller 30·4 % (or 171 in 563) of students who paid their most recent utility bill were food insecure (X^2^ = 27·5, 1 df, *P* < 0·05). As might be expected, nearly 70 % (or 21 in 30) of students who ‘strongly agreed’ they had trouble paying their accommodation reported they were food insecure compared with 16·8 % (or 31 in 184) of students who were food insecure but ‘strongly disagreed’ that they had trouble paying for their accommodation (X^2^ = 108·8, 4 df, *P* < 0·05).

Finally, as noted, we found that students who reported being significantly ill during the first weeks of the pandemic were much more likely to report being food insecure. During the pandemic, 43·9 % (or 79 in 180) of students who were ill reported that they were food insecure whilst 29·4 % (or 130 in 442) of those who were not ill reported being food insecure (X^2^ = 12·0, 1 df, *P* < 0·01).

### Multivariate results

Logistic regression simultaneously estimated OR for all predictors of food insecurity in the UK student sample. When analysing sample data using logistic regression, we used listwise deletion. As a result, the multivariate analysis examined *n* 555 participants who provided us with answers to all questions used to create the variables included in Table 1. The results of the analysis are presented in Table 2. The table provides the OR and 95 % CI for those ratios. Table 2 explains an estimated 28 % of the variance in student *food insecurity* (i.e. Cox-Snell *R* ^2^) suggesting the model is reasonably specified.

**Table 2:** Multivariate logistic regression model for the OR of being food insecure, October 2020

	OR	95 % CI	*P* value
Student demographics			
Gender (*v*. Male)			
Female	0·96	0·62, 1·49	0·82
Third Gender/Non-Binary/Other	1·12	0·28, 6·13	0·90
Ethnicity (*v*. White)			
Asian	0·66	0·33, 1·30	0·23
Black	0·42	0·15, 1·21	0·11
Mixed	1·23	0·46, 3·25	0·67
Other	1·61	0·54, 7·29	0·54
Age (in Years)	1·01	0·97, 1·05	0·58
International student (*v*. home student)	1·18	0·52, 2·68	0·62
Part time student (*v*. full time student)	1·42	0·63, 3·18	0·68
Year of study	0·91	0·76, 1·09	0·40
Living arrangements (*v*. living alone)			
Students	1·24	0·63, 3·18	0·55
Family	0·85	0·40, 1·83	0·68
Non-relative	1·11	0·13, 9·80	0·93
Economic factors			
Past free school meals (*v*. no FSM)	1·52	0·93, 2·50	0·09
Employed (*v*. not employed)	0·99	0·61, 1·63	0·99
Financial aid from student loans (*v*. no loans)	1·86	1·03, 3·35	0·04^*^
Unable to pay utilities (*v*. paid)	3·09	1·58, 6·77	0·05^*^
Difficulty paying accommodation	1·87	1·58, 2·22	< 0·01^*^
University provision			
Food parcel/box (*v*. no parcel/box)	0·80	0·32, 2·00	0·64
Pandemic impact			
COVID-19 positive (*v*. not positive)	0·97	0·30, 2·52	0·81
Significant illness (*v*. no sig illness)	2·16	1·37, 3·41	< 0·01^*^
Constant	0·05		

*n* 555.

-2LL = 585·06.

Pseudo R2 = 0·18.

* Reject the null hypothesis, *α* level = 0·05.

Turning to the results in Table 2, we found little evidence that *gender, ethnicity, age, student status* or *living arrangements* matter when it comes to food insecurity, when economic and pandemic factors are controlled. Whilst student demographics were not statistically significant indicators of food insecurity, economic factors did stand out as potentially important factors in predicting food insecurity. For instance, relying on financial aid from student loans (*v*. not relying on financial aid from student loans) increased the odds of being food insecure by a factor of 1·9; and being unable to pay your utility bill (*v*. paying your utility bill) increased the odds of being food insecure by a factor of 3·1. Each increase in a category of agreement that accommodation is difficult to pay was associated with an increase in the odds of being food insecure by a factor of 1·9 (*P* < 0·05). We also discovered that being ill increased the odds of being food insecure by a factor of 2·2.

Results for ordinary least squares regression where affirmative responses to the six items AFSSM were used as the outcome variable were similar in that the same economic factors and illness variable were statistically significant with two exceptions (see Appendix A). Receiving a free school meal increased the predicted number of affirmative answers to the six items of the AFSSM by 0·62 (*P* < 0·01); being unable to pay utilities increased the number of affirmative answers by 1·12 (*P* < 0·01) and each category increase in difficulty paying for accommodation increased the number of affirmative answers on the scale by 0·50 (*P* < 0·01). We also found that significant illness increased the predicted number of affirmative answers to the six items of the AFSSM by 0·57 (*P* < 0·01). However, students who received loans to pay for university no longer had different levels of food insecurity than those who did not receive loans (*P* = 0·06).

## Discussion

The results within the present study highlight that a range of economic factors were significantly associated with UK student food insecurity during the COVID-19 pandemic. Unlike prior studies of student food insecurity^(26,27,31,32,35,36)^, few student demographic predictors were associated with food insecurity in the analyses. Thus, the results are unique in that demographic factors have been found to be important in other non-pandemic settings.

Students who were in receipt of loans and students who reported difficulties paying for household utilities and accommodation fees were more likely to be food insecure than those not facing these challenges. As a result, variables that indicated students were facing economic hardship played a large role in their food insecurity status in the UK during the COVID-19 pandemic. This result was not unexpected as economic hardship and reliance on financial aid from student loans is often associated with food insecurity^(26,42)^. Being from a low-income household and holding personal debt, as is the case for many students taking out loans during university, have been shown to significantly relate to poorer financial circumstances, particularly if their family cannot offer additional financial support^(30)^. Moreover, those in receipt of financial aid from student loans often have a higher likelihood of food insecurity^(30,31)^, and those without the financial capabilities to pay their higher education fees outright may be less able to pay for other resources, such as food and bills^(51)^, or need to prioritise their finances to pay for fixed bills such as utilities and accommodation over shopping for food^(42)^. These previously recognised challenges, alongside specific challenges resulting from the COVID-19 pandemic and heightened home energy consumption during lockdown^(52)^, may have contributed further to the findings of the current study.

Unexpected costs, both for students and their families, during the pandemic may have also contributed to the lack of significant findings regarding students’ living arrangements; opposing previous literature which typically demonstrates students who live with family to be most food secure^(5)^. In other words, restricted family household income alongside high energy and food prices may have decreased the ‘home advantage’. Moreover, the present study only asked participants where they lived during term-time, and it is unclear whether participants stated where they typically live during term-time, or where they were living during the COVID-19 pandemic. As living arrangements are strong predictors of student food insecurity in many US-based studies^(5,39)^, this factor should continue to be studied in future research regarding food insecurity in higher education in the UK.

Whilst testing positive for COVID-19 was not associated with levels of student food insecurity, students who reported significant illness during the pandemic in the present study were more likely to be food insecure. This finding supports prior literature that has identified a relationship between poor physical health and high food insecurity^(43,53)^ and a relationship between food insecurity and poor student mental health, including anxiety and depression in an international context, both with and without the influence of COVID-19^(42,43)^. Whilst the present study did not investigate specific illness, it is novel in investigating self-reported illness in UK students during the COVID-19 pandemic and demonstrating a significant association between levels of student food insecurity and overall illness. Thus, whilst the findings from the present study provide a novel addition to the literature in this area, it is unclear whether the self-reported illnesses were associated with COVID-19, or other types of physical or mental illness.

Similarly, the results within the present study demonstrate, at the bivariate level, a significant decrease in food insecurity across years at university; with the highest rates of food insecurity identified for foundation year students, and the lowest levels for students within their 4th year of study or undertaking a PhD programme. Previous literature similarly demonstrates high levels of food insecurity for students who are new to university^(54,55)^. However, the sample size of foundation year students within the present study was small, thus future research should conduct similar work with a larger sample size to determine whether these findings are replicable in a similar UK context. It is important to note that the findings also showed no evidence that distributing food parcels/boxes to HE students affected household food insecurity; suggesting that alternative approaches, such as cash-based models,^(56)^ should be explored by universities.

Many of the other demographic variables investigated within the present study, including gender and ethnicity, were not found to significantly relate to student food insecurity. This is unexpected, as previous research broadly demonstrates significant differences in these demographic factors on levels of food insecurity, typically depicting higher levels of student food insecurity for females than males^(57)^ and for students of a non-white ethnicity^(58)^. The COVID-19 pandemic resulted in the same restrictions nationally, regardless of demographic characteristics. However, it is unclear whether the COVID-19 pandemic altered these differences based on demographic characteristics or whether they were not prevalent in the current study sample at all. It is also important to highlight that the current study is cross-sectional in nature and therefore cause and effect cannot be inferred from the data collected. Nevertheless, prior research shows support for many of the present study’s findings.

In conclusion, the present study provides novel findings regarding the factors associated with student food insecurity within the UK during the COVID-19 pandemic. Similar to previous research, the findings suggest that a range of economic factors, including receiving a student loan, and finding difficulty paying utilities and accommodation bills, are significantly associated with higher levels of food insecurity. Moreover, reporting a significant illness during the COVID-19 pandemic was also related to increased levels of food insecurity for higher education students within the UK. Refuting previous literature, however, other demographic factors, including gender and ethnicity, were not found to significantly relate to food insecurity. Importantly, food insecurity was prevalent across all groups investigated, which has implications for how government and universities would address this issue in the context of future pandemics, especially in relation to student finances and the distribution of food parcels.

## Appendix

**Appendix A:** Ordinary Least Squares Regression Model for the Number of Affirmative Responses to the Six-Item Adult Food Security Survey Module, October 2020

	Unstandardized coefficient	95 % CI	*P* value
Student demographics			
Gender (*v*. Male)			
Female	−0·06	–0·37, 0·25	0·69
Third Gender/Non-Binary/Other	0·26	–0·96, 1·49	0·68
Ethnicity (*v*. White)			
Asian	−0·27	–0·73, 0·19	0·25
Black	−0·42	–1·09, 0·24	0·21
Mixed	0·13	–0·58, 0·83	0·73
Other	−0·48	–1·62, 0·66	0·41
Age (in years)	0·01	–0·02, 0·04	0·59
International student (*v*. home student)	0·02	–0·53, 0·57	0·94
Part time student (*v*. full time student)	0·19	–0·37, 0·76	0·51
Year of study	−0·06	–0·19, 0·06	0·34
Living arrangements (*v*. living alone)			
Students	−0·23	–0·78, 0·32	0·42
Family	−0·49	–1·03, 0·05	0·07
Non-relative	0·41	–1·05, 1·87	0·58
Economic factors			
Past free school meals (*v*. no FSM)	0·62	0·26, 0·99	< 0·01*
Employed (*v*. not employed)	−0·19	–0·55, 0·16	0·28
Financial aid from student loans (*v*. no loans)	0·38	–0·02, 0·77	0·06
Unable to pay utilities (*v*. paid)	1·12	0·57, 1·67	< 0·01*
Difficulty paying accommodation	0·50	0·38, 0·63	< 0·01*
University provision			
Food parcel/box (*v*. no parcel/box)	0·12	–0·57, 0·8	0·73
Pandemic impact			
COVID-19 positive (*v*. Not Positive)	−0·52	–1·3, 0·25	0·19
Significant illness (*v*. No Sig Illness)	0·57	0·24, 0·9	< 0·01*
Constant	0·10		

*n* 555.

*F* (21, 533) = 8·85.

Adjusted *R*^2^ = 0·22.

* Reject the null hypothesis, *α* level = 0·05.
